# Stop Codon Polymorphisms in the Human SLC9A1 Gene Disrupt or Compromise Na^+^/H^+^ Exchanger Function

**DOI:** 10.1371/journal.pone.0162902

**Published:** 2016-09-16

**Authors:** Xiuju Li, Aruna Augustine, Shuo Chen, Larry Fliegel

**Affiliations:** Department of Biochemistry, University Alberta, Edmonton, AB T6G 2H7, Canada; Emory University School of Medicine, UNITED STATES

## Abstract

The NHE1 isoform of the mammalian Na^+^/H^+^ exchanger is a ubiquitous plasma membrane protein that regulates intracellular pH in mammalian cells by removing one intracellular proton in exchange for one extracellular sodium. Deletion of the NHE1 gene (SLC9A1) affects the growth and motor ability of mice and humans but mutations and polymorphisms of the gene are only beginning to be characterized. NHE1 has a cytosolic C-terminal regulatory tail of approximately 315 amino acids and a 500 amino acid membrane domain. We examined the functional effects of three human stop codon mutations at amino acids 321, 449 and 735 in comparison with a mutant that had a shortened tail region (543 stop codon). The short mutants, 321, 449 and 543 stop codon mutant proteins, lost NHE1 activity and expression, and did not target to the plasma membrane. Protein for these short mutants was more rapidly degraded than the wild type and 735 ending proteins. The 735 terminating mutant, with the membrane domain and much of the cytosolic tail, had reduced protein expression and activity. The results demonstrate that early stop codon polymorphisms have significant and deleterious effects on the activity of the SLC9A1 protein product. The 735-NHE1 mutant, without the last 80 amino acids, had more minor defects. Surprisingly, retention of a proximal 43 amino acids adjacent to the membrane domain did little to maintain NHE1 expression, targeting and activity.

## Introduction

In mammalian cells, a key protein in the maintenance of pH homeostasis in both normal and neoplastic cells is the Na^+^/H^+^ exchanger isoform one (NHE1). NHE1 is an integral plasma membrane protein consisting of a 500 amino acid membrane domain that facilitates ion movement and a cytoplasmic C-terminal domain of 315 amino acids that regulates activity. NHE1 has been shown to play an important role in promoting cell growth, proliferation, differentiation, and apoptosis. In Na^+^/H^+^ exchange, one intracellular proton is extruded in exchange for a single extracellular sodium ion. Transport is driven by the inward transmembrane Na^+^ gradient. Human NHE1 is the product of the SLC9A1 gene and is ubiquitously expressed in mammalian cells. NHE1 is allosterically regulated. At low intracellular pH (pH_i_), protons allosterically activate NHE1, facilitating proton extrusion and a return to homeostatic pH_i_, at which point the NHE1 protein becomes inactive. NHE1 is critical in several human diseases. It plays an important casual role in the myocardial damage that occurs during ischemia and reperfusion and has been implicated as a mediator of heart hypertrophy. Additionally, NHE1 plays an important role in several types of cancer including acting as a trigger in breast cancer metastasis [1–3].

Natural mutations in the NHE1 gene (encoded by SLC9A1) have not been well characterized, particularly in humans. Mice with disruptions in the NHE1 protein exhibit ataxia, growth retardation and recurrent seizures by 2 to 3 weeks of age. They also exhibit early death [4, 5]. A similar phenotype occurs in humans. A novel and severe human mutation in the NHE1 gene has recently been shown to cause ataxia-deafness and the disease Lichtenstein-Knorr syndrome [6]. This indicates that the human SLC9A1 protein is susceptible to mutations that can cause disease.

There have been very few studies investigating genetic variation in the SLC9A1 gene. These polymorphisms are of interest to both population biologists and functional geneticists [7]. Stop codon polymorphisms (SCPs) in particular, have not been studied. The functional consequences of SCPs that result in premature termination of the protein could be severe because of loss of the carboxyl terminal protein domain, which may affect protein function. Alternatively, there may be a loss or dissociation from 3′ untranslated region regulatory elements. Alteration of the relative position of the stop codon can also result in degradation of the transcript and reduction in protein expression levels. Transcripts carrying premature termination codons may undergo nonsense-mediated decay that results in their loss that occurs when stop codons occur more than 50 bases prior to the final exon-exon junction [7–9].

One recent summary of human genetic variation is the 1000 Genomes Project [10]. Here the genome of a large number of individuals was sequenced and the location of many polymorphisms recorded. In the SLC9A1 gene we noted several polymorphisms with stop codons at amino acids 321, 449 and 735. In the present study, we examined the effect of these mutations on the expression, targeting, and activity of the NHE1 protein and compared them with a truncation of NHE1 at amino acid 543. We demonstrate that the 321, 449 and 543 stop codon mutants are defective in expression, surface targeting, and NHE1 activity and have increased protein degradation rates. To our knowledge, this is the first characterization of the effect of mutant stop codon mutations on the SLC9A1 gene.

## Materials and Methods

### Materials

Sulfo-NHS-SS-biotin for surface labeling was purchased from Thermo Fisher (Waltham, MA) and synthetic DNA for mutagenesis was from IDT (Coraiveille, Iowa, USA). 2’7-bis(2-carboxyethyl)-5(6) carboxyfluorescein acetoxymethyl ester (BCECF-AM) for pH_i_ measurement was from Molecular Probes, Inc. (Eugene, Oregon, USA). Lipofectamine^™^ 2000 reagent for transfection was from Invitrogen Life Technologies (Carlsbad, California, USA). Cyclohexamide was Santa Cruz Biotechnology Inc, (Dallas, TX, USA). Other chemicals were of analytical grade and were acquired from Fisher Scientific, BDH (Toronto, Ontario, Canada) or Sigma-Aldrich (St. Louis, Missouri, USA). The plasmid pYN4+ was used to express cDNA for the human NHE1 protein. It has a HA (hemagglutinin) tag, as described earlier [11].

### Cell culture and stable transfection

To characterize the activity of the wild type vs. mutant Na^+^/H^+^ exchanger we used AP1 cells. This mutant cell line is derived from Chinese hamster ovarian cells and does not express its own protein NHE1 [12]. LIPOFECTAMINE^™^ 2000 reagent was used to stably transfect the AP1 cells [12]. The pYN4+ plasmid contains a neomycin resistance cassette that allows selection of stably transfected cells using G418 antibiotic. Cell lines were regularly re-established from frozen stocks at between passage numbers 5–11. Results are typical of at least two stable cell lines made independently. We used the wild type and mutant plasmids for transient transfections of AP1 cells using LIPOFECTAMINE^™^ 2000 Reagent in some experiments [12].

### Plasmid construction

The plasmid pYN4+ has the cDNA of human NHE1 followed by a HA tag. To engineer premature stop codons in the NHE1 sequence we inserted an XhoI site before the HA tag by site directed mutagenesis using these primers (F-GAGGGAGAACCGTTCTTCCCCAAGctcgAGgatgacGGCCGCATCTTTTACCC R-GGGTAAAAGATGCGGCCgtcatcCTcgagCTTGGGGAAGAACGGTTCTCCCTC). A second round of mutagenesis was used to change a designated amino acid to a stop codon. This mutagenesis also inserted a second XhoI site designed such that the intervening XhoI-XhoI piece could be deleted with an XhoI digestion. After digestion the vector was re-ligated to get the appropriate plasmid now shortened at the indicated amino acid and still containing an HA tag.

The primers for different stop mutants are listed in Table 1. DNA sequencing confirmed the mutation and the fidelity of DNA amplification. NHE1 proteins shortened at amino acids 321, 449, 543 and 735 were designated 321-NHE1, 449-NHE1, 543-NHE1 and 735-NHE1 respectively.

**Table 1:** Oligonucleotides used for site-directed mutagenesis (forward primer).

Mutation	Oligonucleotide sequence with the Xhol site in lower case
321-NHE1	5′- GGGGTCATCGCAGCCTTCACCTCCctcgagACCTCCCACATCCGGGTCATCGAG-3′
449-NHE1	5′- GTGAAGCTGACCCCCAAGGACCtcgagATCATCGCCTATGGGGGCCTG-3′
543-NHE1	5′- GAAGACATCTGTGGCCACTACGGTCtCgAgCACTGGAAGGACAAGCTCAACCGG-3′
735-NHE1	5′- GAGTCTGTGGACCTGGTGAATctcGAGCTGAAGGGCAAAGTCTTAGGG-3′

### Cell surface expression

Targeting of the NHE1 protein to the cell surface was measured by labeling cell surface proteins with sulfo-NHS-SS-biotin [13]. The cell proteins were solubilized and cell surface proteins (and the cell surface Na^+^/H^+^ exchanger) were removed using immobilized streptavidin resin. Western blotting for immunoreactive (HA-tagged) NHE1 protein was used to probe for NHE1 in total cell proteins and probing for NHE1 protein remaining after removal of cell surface proteins with streptavidin-agarose [14, 15]. The levels of the NHE1-HA immunoreactive protein present on the western blots were estimated using Image J 1.35 software (National Institutes of Health, Bethesda, Maryland, USA). We have found that it not possible to reproducibly elute proteins bound to immobilized streptavidin resin because of the high affinity of streptavidin for biotin. Quantification was of both the upper and lower HA-immunoreactive species of the NHE1 protein was using Image J and the percentage of the protein targeting on the cell membrane was calculated with the equation: (Total-unbound)/Total x 100%.

### SDS-PAGE and immunoblotting

We examined the levels of NHE1 in stably or transiently transformed AP1 cell lines via immunoblotting against the HA tag of the NHE1 protein. Samples of cell lysates were separated on SDS-PAGE (10%) gels and were transferred to nitrocellulose. One hundred μg of protein of control and experimental lysates (321-NHE1, 449-NHE1, 543-NHE1 and 743-NHE1) were run in triplicate for quantification. Protein concentrations were measured with a BioRad D/C^™^Protein Assay kit. The primary antibody to identify tagged NHE1 was anti-HA monoclonal antibody. The secondary antibody was peroxidase-conjugated goat anti-mouse antibody (Bio-Can, Mississauga, Canada). Protein reactivity was detected using X-ray film via the Amersham enhanced chemiluminescence western blotting and detection system. Quantification of protein expression from exposed X-ray films of western blots was using Image J 1.35 software (National Institutes of Health, Bethesda, MD, USA).

### Intracellular pH measurement

To measure pH_i_ cells were grown to approximately 80%–90% confluence on glass coverslips. BCECF was loaded into cells as described earlier [16]. Fluorescence was measured using a PTI Deltascan spectrofluorometer and Na^+^/H^+^ exchanger activity was measured after an acute acid load was induced, as described earlier [16]. Ammonium chloride (50 mmol/L × 3 min) addition followed by removal was used to induce the acute acidosis. The first 20 s of recovery in NaCl-containing medium was measured as ΔpH/s. This rate of recovery was measured in the wild type NHE1 protein and compared with the rate of recovery in the shortened NHE1 mutant proteins. Calibration of pH_i_ fluorescence was done for every sample using nigericin as described earlier [12]. Results are shown as the mean ± S.E. of at least 8 experiments and statistical significance was determined using the Wilcoxon-Mann Whitney test. In some experiments we acidified cells to varying pHi levels, the ammonium chloride concentration was reduced to various concentrations from 50 mM to 5 mM as described earlier [17].

### mRNA and Protein Degradation of Wild Type and Mutant NHE1 Proteins

To examine mRNA degradation RNA was extracted from various stable cell lines using the RNeasy Minikit (Qiagen) according to the manufacturer's protocols. Total RNA (1 μg) was reversed transcribed from the various cells lines to cDNA using iScript RT Supermix (BioRad). Quantitative RT-PCR was then performed using the iQ SYBR Green Supermix (BioRad) in a Rotor-Gene RG3000 cycler (Corbett Research) with 40 cycles per sample. Cycling temperatures were: denaturing, 95°C, annealing, 55°C, and extending, 72°C. The primers were designed to amplify from a common reverse primer for wild type NHE1 and mutants (RevNHEQPCR GCGGCCAATTCTAGCACTGAG) and a forward primer designed to give a PCR product the same size, based on the appropriate sequence of wild type and each mutant protein, (Wild type, 5’-GCATCATGATGCGGAGCAAG-3’; 735, 5’-GAAGATCAACAACTACCTG-3’; 543, 5’-CTTCACCGTCTTTGTGCAG-3’; 449, 5’-CCTCGGCGTCTCCACGGTG-3’; and 321, 5’-CGCCGTCACTGTGGTCCTG-3’. Primers for CHO cell GAPDH (Forward 5’-CAGGTTGTCTCCTGCGACTT-3; Reverse 5’-GGGGTCTGGGATGGAAACTG-3’ were used to amplify control transcript. Differences in gene expression are presented as fold changes normalized to GAPDH expression. Cumulative data from at least three independent experiments are shown. Cells were treated with 1.25 μM actinomycin D (or DMSO) for up to 24 hours. Results from one time point after 8 hours are shown.

To examine NHE1 protein stability wild type and mutant NHE1-containing cell lines grown and were treated with 50 μM cyclohexamide for up to 8 hours. Cell lysates were examined for NHE1 protein expression by western blotting versus the HA tag on the NHE1 protein. Quantification of NHE1 protein levels was using Image J 1.35 software as described above.

### Immunofluorescence

Cells were grown as described above on coverslips and were gently rinsed with phosphate buffered saline (PBS) two times. They were fixed with 4% paraformaldehyde in PBS for 15 min at room temperature and washed twice with PBS for 10 minutes. 50 mM ammonium chloride in PBS was added for 10 min and this was removed by washing 3 times with PBS. Cells were permeabilized for 10 min with 0.2% Triton X-100 and then washed with PBS for 10 min. Cells were blocked with 5% goat serum (blocking buffer) for 45 min and then incubated with the primary antibody in the same buffer for a maximum of 1 hr at room temperature. Cells were washed with blocking buffer two times and then once with PBS. Secondary antibody in blocking buffer was added for 1 hr in the dark and this was removed by washing three times with PBS. Staining with DAPI (300 nM) was for 10 min in the dark followed by two rinses with PBS and one with water. Several primary antibodies were used. A monoclonal against HA (for HA tagged NHE1) used at 1:200 dilution. For some experiments a mouse monoclonal antibody against the distal region of the NHE1 cytoplasmic tail was obtained from BD Biosciences and used at a 1:200 dilution. A rabbit polyclonal anti HA antibody was obtained from Santa Cruz and used at a dilution of 1:200. A secondary goat anti-mouse antibody (Jackson Immunoresearch Labs) was used at 1:200 and was conjugated to Alexa fluor 488. A secondary goat anti-rabbit antibody (from Rockland Inc.) conjugated to Alexa 647 (Atto647) was used at a 1:200 dilution. Images were obtained with a Leica SP5 confocal laser scanning microscope with a 63 X objective. Lasers and laser intensity were 405 Diode for DAPI, 10% detection at 435–465 nm. Argon for Alexa 488, 20% at 510–560 nM. HeNe for Alexa 647, 33% at 640–700 nm. Images were obtained using Leica Application suite-AF software (Leica Microsystems) and processed identically.

## Results

**Fig 1:**
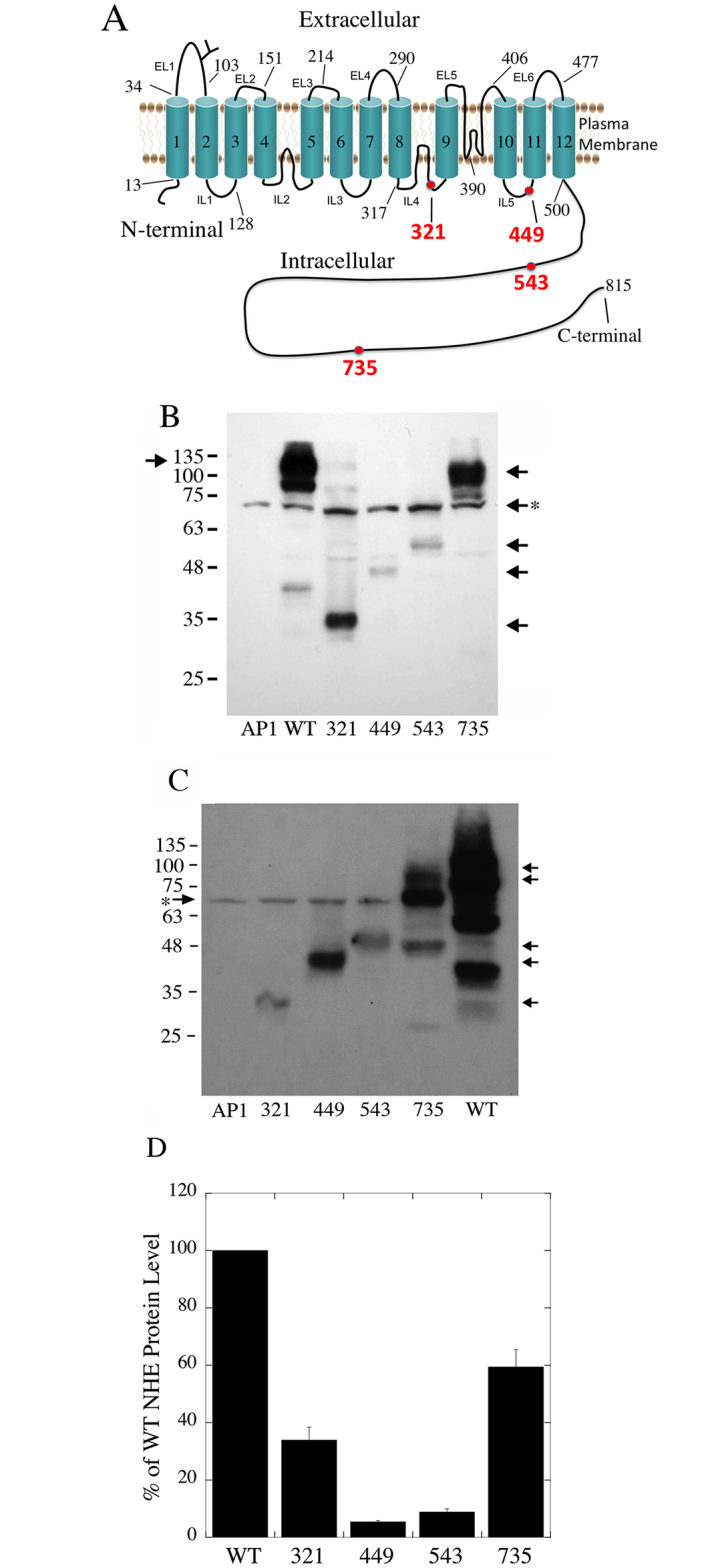
Wild type (WT) NHE1 protein expression and characterization. **A**, Model of the NHE1 protein within the plasma membrane [18]. The approximate position of the shortened NHE1 proteins is indicated in red. B, Western blot of whole cell lysates of stably transfected cell lines. Mutants are with NHE1 protein terminated at amino acid, 321, 449, 543 and 735 as indicated. AP1 indicates mock transfected AP1 cells. Right arrows indicate the HA immunoreactive band of the 735, 543, 449 and 321 proteins. Right arrow with asterisk indicates non-specific immunoreactive band present in all cell lysates. Left arrow indicates HA immunoreactive band of WT NHE1 protein. **C**, Western blot of whole cell lysates of transiently transfected AP1 cells. Mutants are with NHE1 protein sequence terminated at amino acid, 321, 449, 543 and 735 as indicated. Right arrows indicate the HA immunoreactive bands as above. Left arrow with asterisk indicates non-specific immunoreactive band present in all cell lysates. **D**, Summary of levels of expression of stably expressed NHE1 protein in AP1 cells. Numbers are percent of the expression levels of mutants in comparison to wild type protein, mean ± S.E. n = at least 3 determinations.

An analysis of the data of the 1000 genomes project revealed a number of polymorphisms in the SLC9A1 gene. Three of these were stop codons. They were mutations of amino acids 321, 449 and 735 to stop codons (ID’s rs150618776, COSM133924 and COSM126929 respectively). All were single base pair mutations two being cag to tag mutations and one being a gaa to taa mutation for amino acid 735. For comparative purposes, we also made a mutant NHE1 protein that ended at amino acid 543. The approximate position of the mutations in the NHE1 protein is shown in Fig 1A. Amino acid 321 and 449 are within the transmembrane domain and amino acid 543 and 735 are within the C-terminal regulatory tail [18]. The protein ending at amino acid 543 contains approximately 43 amino acids distal to the transmembrane segment XII, which is the final transmembrane segment of the membrane domain. The 43 amino acids contain binding sites for CHP1 (calcineurin homologous protein isoform 1) that is purported to be important in targeting of the NHE1 protein to the plasma membrane [19, 20].

The four mutant NHE1 mutations were made and confirmed by DNA sequencing. Stable cell lines of these and the control were made in AP1 cells, that are a derivative of CHO cells that are devoid of their own NHE1 protein. We then characterized activity, targeting and expression levels of these proteins relative to that of the wild type NHE1 protein. All constructs had an HA tag that we have earlier shown does not affect NHE1 activity [16]. Western blotting was used to examine the expression levels of the mutant and wild type proteins. Fig 1B–1D shows examples of the results and a summary of expression levels. Fig 1B illustrates the expression levels of the mutant and wild type protein. The full-length NHE1 protein is expressed as a doublet. This is made of a fully glycosylated NHE1 protein and a partially glycosylated or de-glycosylated protein [13, 21]. As expected, the full-length wild type protein was larger than the other shortened NHE1 proteins. The 735-NHE1 protein was slightly reduced in size compared to the wild type protein, and still showed evidence of heterogeneous size. The other shortened proteins appeared largely as a single molecular weight species though there was some evidence of degradation in most of the samples. Fig 1C illustrates a similar experiment though the samples came from cells transiently transfected with DNA for the NHE1 proteins. The same general trends were present with the wild type protein expressed at a higher level than the mutant proteins. The 735-NHE1 protein again showed evidence of heterogeneous size and was also expressed at a higher level than the other mutant proteins. In this case there was more evidence of degradation of the NHE1 protein. This may be a result of a higher level of expression of NHE1 in the transiently transfected cells and may also partially reflect a longer exposure time of the film that was done to illustrate the low level of the 321-NHE1 protein. Fig 1D summarizes the relative expression levels of the stably expressed NHE1 proteins. All the mutants were reduced relative to the wild type, with the 735-NHE1 protein having the highest levels of expression. Surprisingly, there was not a direct linear correlation with length of the NHE1 protein vs. expression levels. The 321-NHE1 protein was expressed at higher levels than the 543-NHE1 and 449-NHE1 proteins.

**Fig 2:**
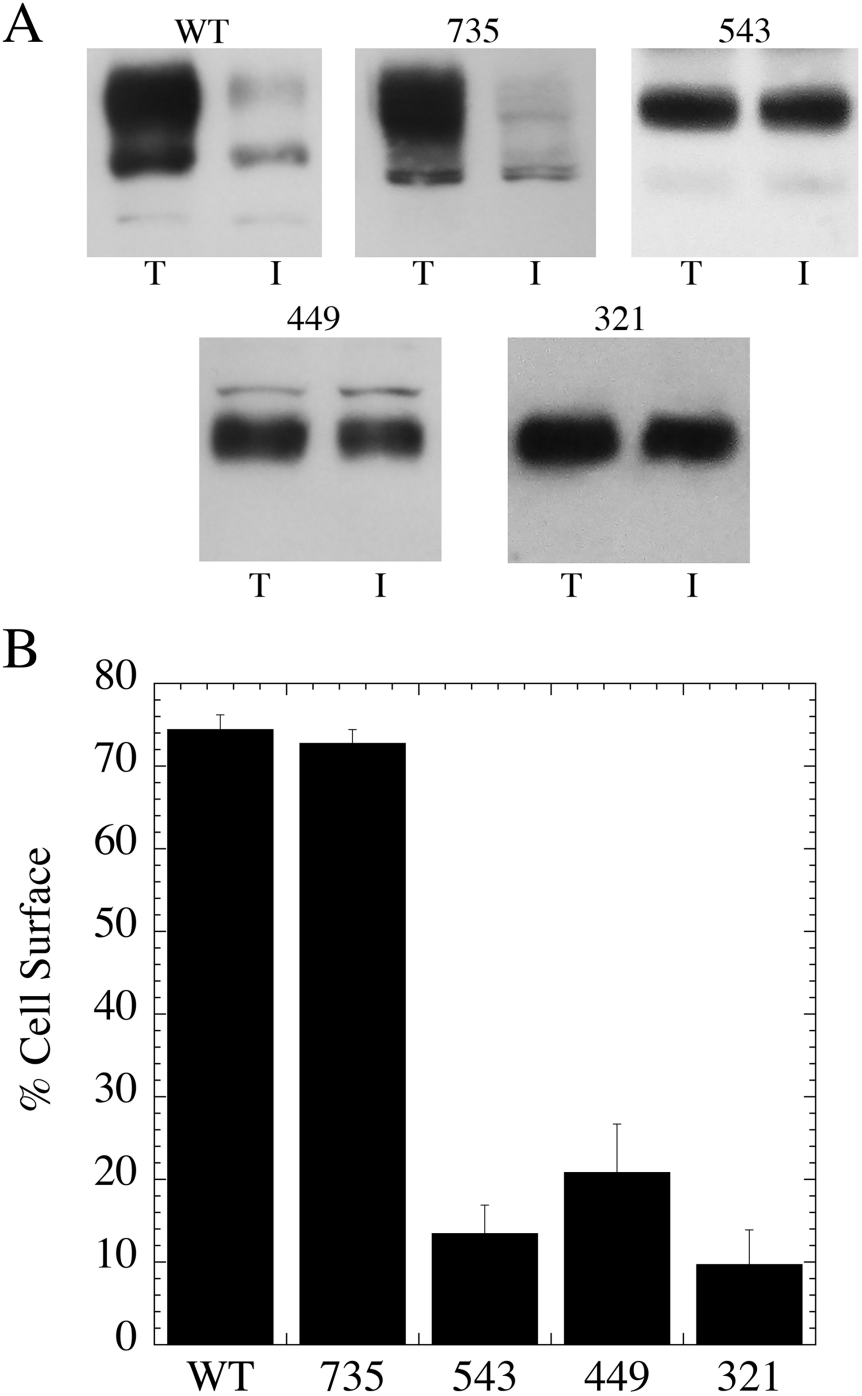
Surface localization of wild type (WT) and mutant NHE1 proteins in AP1 cells. **A**, example of results and **B**, mean ± the S.E. n = at least 3 determinations. After cell surface biotinylation, equivalent amounts of a total cell lysate (T) and an unbound intracellular lysate (I) were examined by western blotting with anti-HA antibody to identify NHE1 protein. WT and mutant cell lines (321, 449, 543 and 735 as indicated) are cell lysates from cell lines stably expressing wild type NHE1 and mutant NHE1 proteins respectively. The % targeted to the cell surface is indicated.

Shortening of the NHE1 protein could result in mistargeting with a lack of expression on the cell surface. Other mutations of NHE1 have had this effect [12, 16]. To determine if cell surface targeting was affected we examined targeting of the mutant proteins in comparison to that of the wild type NHE1 protein. Cell surface proteins were biotinylated and we examined the fraction of NHE1 protein on the cell surface as described in the “Materials and Methods”. The results (Fig 2A and 2B) showed that only the wild type NHE1 protein and 735-NHE1 targeted well to the cell surface. All of the shorter proteins targeted poorly to the cell surface.

**Fig 3:**
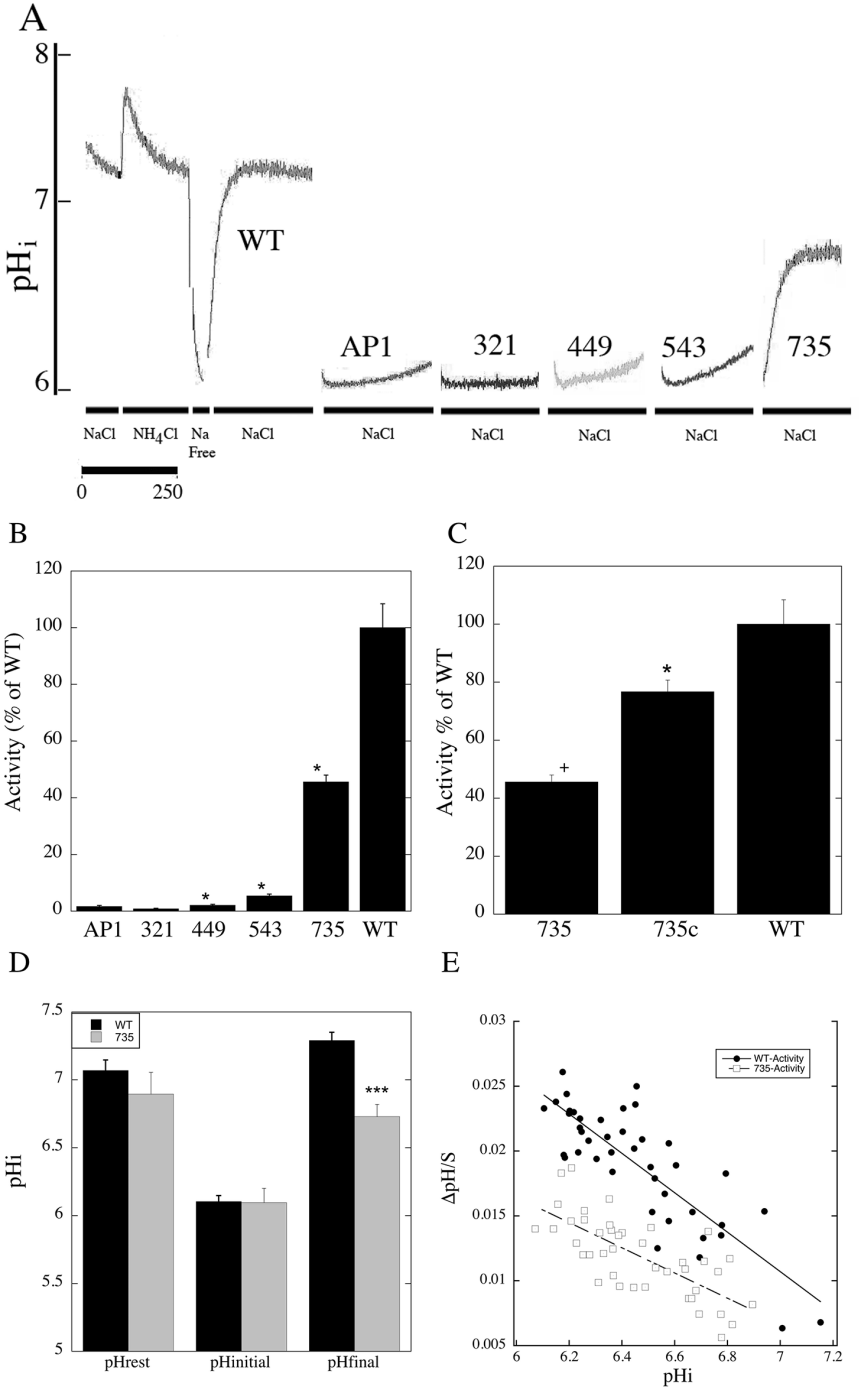
Analysis of Na^+^/H^+^ exchanger activity of wild type (WT) and mutant NHE1 proteins in AP1 cells. Na^+^/H^+^ exchanger activity was assayed in stably transfected AP1 cells grown on coverslips as described above. **A**, Example of NHE1 activity of AP1 cells containing stably transfected wild type NHE1 and NHE1 mutant proteins. For clarity, only the recovery from ammonium chloride induced acidosis is shown for the mutant NHE1 proteins. NH_4_Cl, treatment with ammonium chloride. To induce acidosis following NH_4_Cl treatment there is a brief “Na Free” treatment. NaCl, = recovery period from acidosis in NaCl containing buffer. **B,** Summary of activity of WT and mutant (321, 449, 543 and 735 as indicated) NHE1 proteins in stably transfected AP1 cells. The NHE1 activity was measured after an ammonium chloride prepulse as illustrated in “A”. The initial rate of recovery in NaCl-containing medium was measured as ΔpH/s. Mutants activity are presented relative to that of the wild type protein, which was set at 100%. * indicates significantly different from wild type *P < 0.0001, n>8. The mean value of the wild type NHE1 activity was 0.026 **Δ**pH/s. **C**, Summary of Na^+^/H^+^ exchanger activity of NHE1-735 protein in comparison to wild type NHE1 protein. The 735-NHE1protein activity is displayed as a percent of the wild type (WT). 735c is the activity of the 735-NHE1 protein that has been corrected to the expression levels and surface targeting of the wild type NHE1 protein *P < 0.05, ^+^P < 0.01, n>8. **D**, Resting pH_i_ of wild type and 735-NHE1 protein containing cells at various times prior to and during ammonium chloride induced acidosis. pHrest, resting pHi prior to treatment with ammonium chloride; pHinitial, initial pH_i_ after ammonium chloride treatment and prior to recovery in sodium containing medium; pHfinal, final pH_i_ 3 minutes after recovery from ammonium chloride in sodium containing medium. n>8, ***P < 0.001. **E**, Characterization of Na^+^/H^+^ exchanger activity of wild type and 735-NHE1protein over different intracellular pHs (pH_i_). Cells containing wild type or 735-NHE1 protein were acidified to different levels by addition of varying amounts of ammonium chloride as described in the “Materials and Methods”. The initial rate of recovery was measured and recorded and **Δ** pH/s.

We next examined the Na^+^/H^+^ exchanger activity of the wild type and mutant containing cells using a fluorometric assay. Fig 3A illustrates an example of the wild type NHE1 activity and that of an inactive mutant. Fig 3B summarizes the results. The wild type NHE1 protein displayed robust recovery from acute intracellular acidosis. The NHE1 protein ending at amino acid 735 displayed about 50% of this activity. The 321-NHE1 and 449-NHE1 proteins did not have significant activity while the 543-NHE1 protein displayed only about 5% of the wild type NHE1 protein activity.

We further characterized pH regulation in the AP1 cell line containing the NHE1 protein with the sequence shortened to amino acid 735. In Fig 3C we illustrate the Na^+^/H^+^ exchanger activity of the protein that was corrected for the amount of NHE1 protein expressed and targeted to the cell surface. The results show that the level of activity of this protein is still reduced relative to that of the control.

We compared the intracellular pH of wild type cells with that of the NHE1 protein with the sequence shortened to amino acid 735 (Fig 3D). Both the resting intracellular pH, prior to ammonium chloride treatment, and the degree of acidification induced by ammonium chloride (pHinitial), did not vary between wild type and 735-NHE1 protein containing cells. Cells recovering from an acute acid load induced by ammonium chloride, reach a plateau 3 minutes after recovery begins (i.e. Fig 3A). We therefore examined the resting intracellular pH at this time after recovery from ammonium chloride induced acidosis. The results demonstrate that the shortened 735-NHE1 protein did not return intracellular pH to the same level as the wild type protein. Intracellular pH remained approximately half a pH unit below that of the cells containing the wild type NHE1 protein. To determine the profile of activation of the wild type and mutant 735-NHE1 proteins we acidified the cells to varying intracellular pH with different amounts of ammonium chloride. Wild type NHE1 protein exhibited greater Na^+^/H^+^ exchanger activity at all intracellular pH’s, (Fig 3E) with a notable shift in the activity vs. intracellular pH curve.

**Fig 4:**
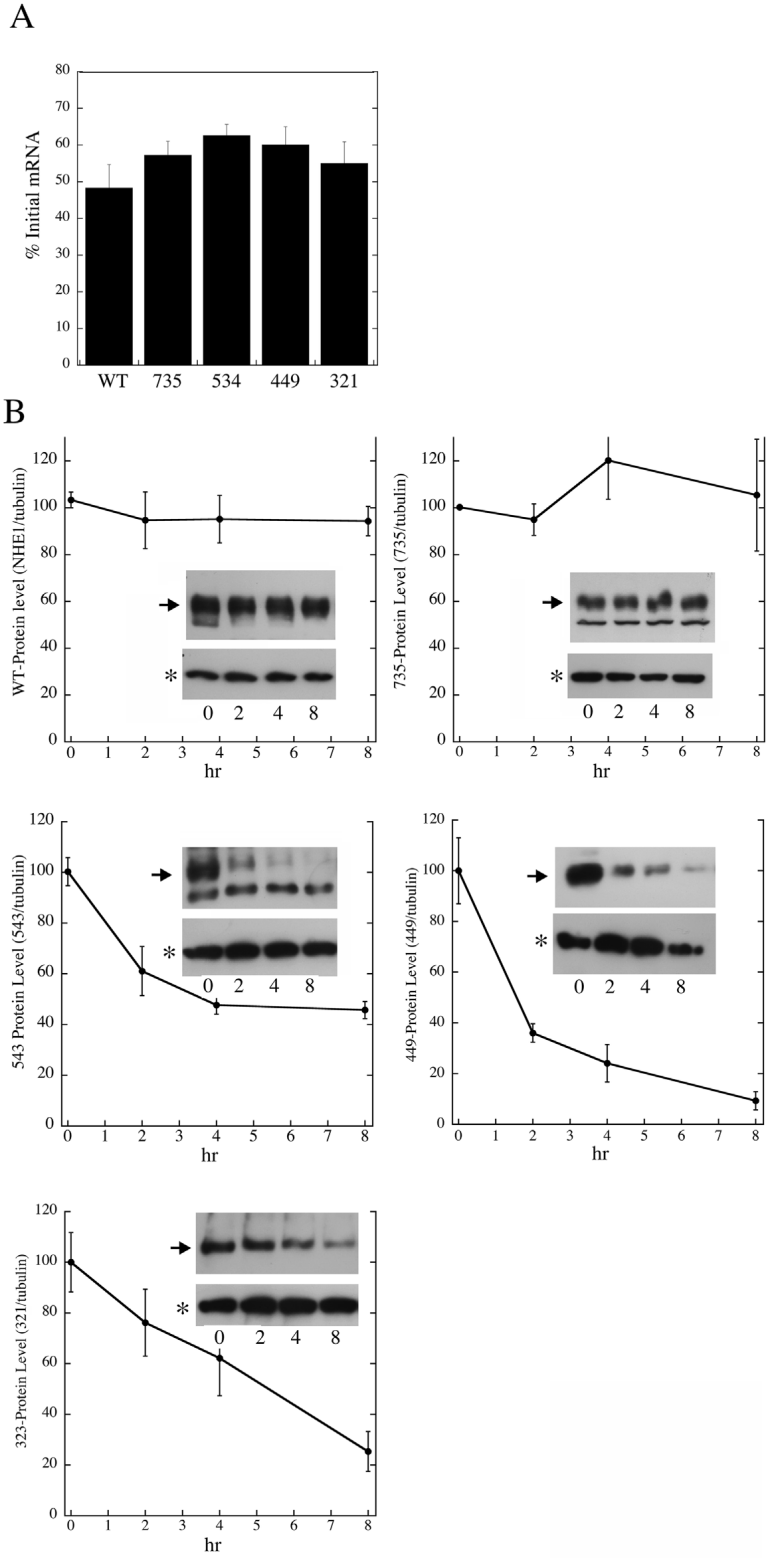
Examination of mRNA and protein stability of mutant and wild type Na^+^/H^+^ exchangers. **A.** mRNA levels of wild type and mutant NHE1 proteins 8 hours after treatment with 1.25 μM actinomycin D. mRNA levels were measured by quantitative RT-PCR. Levels are relative to the initial starting mRNA level prior to treatment and were corrected by the level of GAPDH. Results are mean +/- SE of at least 3 experiments. Individual experiments had 8 replicates. **B.** Protein levels of wild type and mutant NHE1 measured at time 0, (starting time) and up to 8 hours after cyclohexamide (50 μM) addition. Equal amounts of total proteins were loaded in each lane. NHE1 levels were determined by western blotting against the HA tag on the protein. Quantification was via were estimated using Image J 1.35 software. Insets are example western blots showing NHE1 levels (upper panel) in comparison to tubulin levels. Full sized NHE1 protein is indicated with an hour tubulin is indicated with an asterisk. Time points at 0 hr, 2, 4 and 8 are indicated. Results are mean +/- SE of at least 3 experiments.

We examined the degradation of mRNA of the wild type and mutant NHE1 proteins in the stable cell lines to determine if differences in RNA stability could account for the reduced levels of the mutant NHE1 proteins that we found. Cells were treated with actinomycin D to inhibit mRNA biosynthesis and mRNA levels were examined over a period of 24 hours. Results from the 8 hour time point are shown in Fig 4A. The wild type NHE1 mRNA level decreased to about 50% of the starting value 8 hours after actinomycin D treatment. The mutant NHE1 proteins decreased to between 55 and 63% of the initial starting levels of mRNA. There was a slight increasing tendency for the mRNA to be degraded less after 8 hours, centering at the 543 mutant protein, but this was not statistically significant. It was clear however that the mutant mRNA was not degraded more rapidly than the wild type mRNA.

To determine if changes in the length of NHE1 affected the degradation of the NHE1 protein we examined the stability of the wild type and mutant NHE1 protein. Cells were treated with cyclohexamide (50 μM) for varying time periods up to 8 hours. The level of NHE1 protein was determined by western blotting against the NHE1 protein and the level of protein was estimated using was using Image J 1.35 software. The results (Fig 4B) demonstrate that both the wild type protein and the 735-NHE1 protein were relatively stable over the 8 hour time period. In contrast, the remaining 3 shorter mutants were degraded much more rapidly. The level of the 543-NHE1 protein was less than half the starting value while the 449-NHE1 protein and the 321-NHE1 protein were degraded to even lower levels after 8 hours.

**Fig 5:**
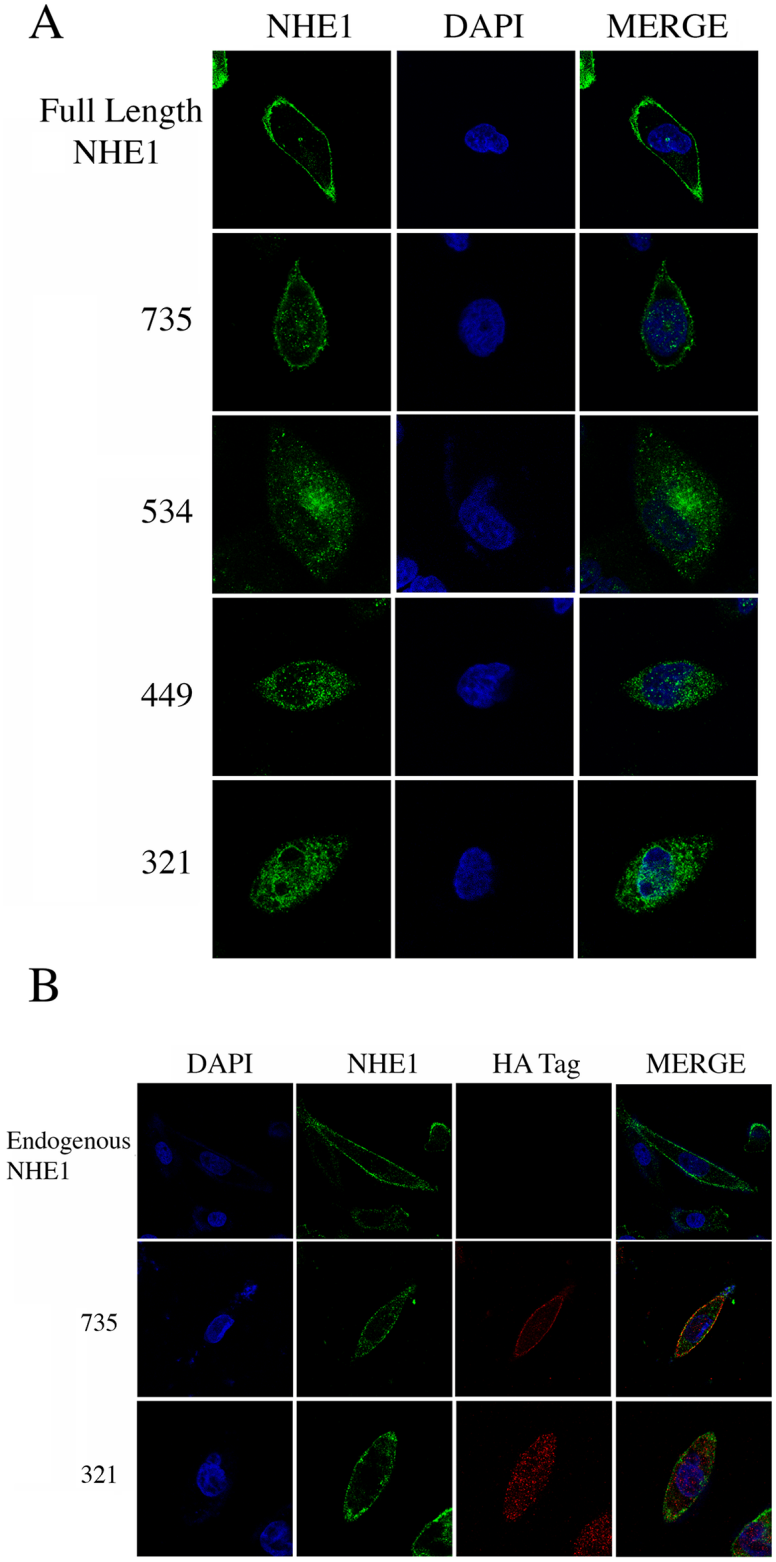
Analysis of NHE1 localization. **A, Localization of wild type and mutant NHE1 proteins in AP1 cells.** Wild type (full length) and mutant NHE1 proteins were expressed in AP1 cells and examined for localization using antibodies against the HA-tag present on each protein. Left column shows the staining for NHE1 protein of the wild type or mutant NHE1 protein. Centre column shows DAPI staining and the right column shows merger of the two images. **B, Effect of co-expression of wild type and mutant NHE1 proteins on protein targeting.** All experiments in this Fig were using CHO cells which posses their own, endogenous NHE1 protein. **DAPI**, column 1, DAPI staining. **NHE1**, column 2, staining with monoclonal anti NHE1 antibody against the distal end of the C-terminal tail. Second antibody was coupled to Alex 488. **HA-tag**, column 3, staining with polyclonal antibody against the HA-tag present on NHE1 proteins transfected into cells. Second antibody was coupled to Alexa 647. **Merge**, column 4, merger of columns 1–3. **Endogenous NHE1**, row 1, CHO cells were not transfected with plasmid expressing NHE1 protein but possess their own endogenous NHE1 protein. **735**, row 2, CHO cells containing their own endogenous NHE1 protein, were transfected with the 735-NHE1 protein. **321**, row 3, CHO cells containing their own endogenous NHE1 protein, were transfected with the 321-NHE1 protein.

We examined the localization of the wild type and mutant NHE1 proteins. Fig 5A shows the localization of the individual proteins. The wild type NHE1 protein and the 735-NHE1 protein are predominantly localized to the cell surface. In contrast, all three shorter proteins have little if any NHE1 protein apparent on the cell surface and are distributed within the cell. DAPI staining revealed the location of nuclear DNA for comparative purposes. These results confirm the results of Fig 2, that the shorter stop codon polymorphism proteins are mistargeted and principally have an intracellular localization.

A second series of experiments examined the effect of expression of NHE1 mutant proteins in combination with wild type NHE1 protein. For these experiments we used wild type CHO cells that have their own endogenous NHE1 protein. In the top row, column two demonstrates the presence of endogenous NHE1 protein using antibody against the C-terminal of the intracellular tail of NHE1. The NHE1 protein was on the cell surface, with some intracellular protein also present. In row two we expressed the 735-NHE1 protein in CHO cells. Antibody against the endogenous NHE1 protein (column 2) does not detect the 735-NHE1 protein as it does not contain the distal region of the tail (see S1 Fig). Column 2, row 2, shows the endogenous NHE1 protein, again with a mostly plasma membrane distribution. Column 3, row 2, shows immunofluorescence against the 735-NHE1 protein, using antibodies against the HA tag. This protein was also mostly present on the cell surface. The merged image of the 735-NHE1 expressing cells (column 4) shows that the endogenous NHE1 and exogenous 735-NHE1 protein co-localize on the cell surface. Row 3 examines the effect of expression of the 321-NHE1 protein in conjunction with the endogenous NHE1 protein. Antibody against the endogenous NHE1 protein again showed a cell surface distribution. Antibody against the HA tag of the 321-NHE1 protein showed an intracellular distribution similar to what was shown in Fig 5A. Merging the images (column 4) showed little overlapping endogenous NHE1 protein and 321-NHE1 protein on the plasma membrane.

## Discussion

Genetic defects in the NHE1 protein have been shown to cause severe effects in mammals, particularly in development. In both mice and humans, a disruption or severe defect in the NHE1 protein causes developmental defects that include growth and developmental retardation, and a variety of neurological features such as ataxia [4–6]. While a complete disruption of NHE1 has severe effects, it is not yet clear if more subtle changes in the genes have significant effects on the protein activity, that could cause milder but significant effects in adult humans. Aside from its involvement in development, the NHE1 gene, SLC9A1, has many very important functions in humans including being involved in heart hypertrophy, in ischemic heart disease, [22], and it is also critical in metastasis of several cancer cell types including breast cancer cells [1, 23, 24]. Thus it seems that polymorphisms in the gene could have a significant effect on its role in these diseases. Stop codon polymorphisms (SCPs) in particular, have not been studied. The functional consequences of SCPs might be severe because of loss of the carboxyl terminal protein domain, which may affect protein function.

For this reason we examined the effects of several recently reported SCPs in the NHE1 gene. Codons were changed to mimic the termination of the NHE1 protein at amino acid 321, 449 and 735. Additionally, for comparative purposes, we made a mutation to mimic a stop codon at amino acid 543. This mutation would retain a small part of the NHE1 cytosolic tail, including a binding site for the CHP protein that is purported to be critical in targeting of the NHE1 protein to the plasma membrane [19, 20]. Analysis of Western blotting confirmed that the shortened proteins were expressed, but at reduced levels compared to the wild type NHE1 protein. This varied in an irregular way with the 735-NHE1 proteins being expressed about 40% less than the wild type protein and the other proteins expressing from 30 to 5% of the wild type protein level. Cell surface targeting was also affected by the mutations though in this case the 735-NHE1 protein was equivalent to the wild type NHE1 protein, while targeting of the other mutants was all greatly reduced. This result was shown in experiments with cell surface biotinylation (Fig 2B) and by immunofluorescence (Fig 5A). A surprising result was that the 543-NHE1 protein was so poorly expressed and targeted, despite the presence of the CHP binding site [19, 20]. Clearly, the presence of this binding site alone is not enough to target NHE1 to the plasma membrane. All the NHE1 constructs were followed by a 39 amino acid long triple HA tag. So that the effect of terminating the NHE1 sequence at amino acid 543 was likely due to omission of some NHE1-specfic sequence and not simply due to shortening the protein. These results suggest that the CHP binding site, while important, is not in itself enough to target the protein to the cell membrane. The 543-NHE1 also had very little Na^+^/H^+^ exchanger activity, less than 5% of the wild type. Certainly this was at least partially due to the very poor targeting and expression of the protein.

Because human mutations may occur as heterozygotes, we were curious to examine if shortened mutant proteins intracellular localization could be affected by the presence of full length NHE1 protein or, conversely, if the presence of the mistargeting mutant proteins could affect the plasma membrane localization of full length NHE1 protein. In Fig 5B we examined the effects of co-expression of the 735-NHE1 and 321-NHE1 proteins with endogenous NHE1 present in CHO cells. We did not find any effects of expression of the 735-NHE1 protein or the 321-NHE1 protein on full length NHE1 protein plasma membrane localization. In addition, there was no apparent effect of expression of endogenous NHE1 on the mutant proteins, in particular, there did not appear to be a rescue of the 321-NHE1 protein to the plasma membrane and it retained an intracellular localization. NHE1 has been demonstrated to be a dimer [25, 26]. However, it appears that the association of NHE1 is not enough to overcome targeting signals. Alternatively, it may be that some of the dimerization motif of the shortened NHE1 proteins may have been removed, preventing their association with the full length NHE1 protein.

The levels of expression of the 321-NHE1, 449-NHE1 and 543-NHE1 protein were varied, but were much lower than the 735-NHE1 protein or the full length NHE1 protein. We examined if these transcripts underwent premature decay due to their premature termination [7–9]. However, we did not find any differences in the degradation of NHE1 transcript in the mutants vs. the wild type protein (Fig 4A). In contrast, the 321-NHE1, 449-NHE1 and 543-NHE1 proteins were much more rapidly degraded in comparison with the wild type and 735-NHE1 protein (Fig 4B). These results demonstrate that the lower level of NHE1 protein of the shorter mutants was at least in part, due to more rapid degradation of the protein.

The 735-NHE1 protein was defective in activity and in regulation of pH_i_. Even after correcting for the lower expression level of the protein, the activity was reduced relative to the wild type Na^+^/H^+^ exchanger. Additionally, after an acute acid load recovery to resting pH_i_ was reduced (Fig 3C and 3D). The amino acids distal to residue 735 include several reported to be important in regulation of the NHE1 protein. This includes phosphorylation sites at amino acids 770 and 771 and 779 and 785. These sites are phosphorylated by Erk and modulate activity of the NHE1 protein [27, 28]. A chaperone [29] and β-Raf [30] have also been found to bind to the C-terminal region of NHE1, which may include the amino acids missing in this region. In addition, there is a binding site for the regulatory protein carbonic anhydrase II in the distal region of the tail that would be missing in this protein. The carbonic anhydrase II binding site is modulated by phosphorylation and affected activity of the NHE1 protein [31]. The region of NHE1 missing distal to amino acid 735 is reported to be disordered in structure, but structure may be induced upon protein binding or with phosphorylation [32, 33]. We have earlier found that phosphorylation in this region of the protein, changes its conformation in a pH dependent manner and affects NHE1 activity. Additionally, this region can interact with upstream regions of the NHE1 tail to regulate activity of the protein [33]. We found that the activity vs. pH_i_ curve of the 735-NHE1 protein was less steep than that of the wild type NHE1 protein and was shifted to the left (Fig 3E) indicative of a lower level of activation at a given intracellular pH. Thus while the 735-NHE1 protein is functional, a stop codon at amino acid 735 alters NHE1 regulation and function. Likely this has significant downstream affects in humans.

Two other short NHE1 proteins, 321-NHE1 and 449-NHE1 were not functional and were not well expressed or targeted. Amino acid 321 and 449 are located in intracellular loops between transmembrane segments 8 and 9, and between 10 and 11 respectively [32, 34]. Premature termination of the NHE1 protein in these locations would result in a protein without four or two transmembrane segments respectively. This would include transmembrane segment eleven, which may be a critical part of the transport mechanism of the protein [14, 32]. Thus, it is not surprising that Na^+^/H^+^ exchanger activity was absent in cells expressing these proteins. Cox et al. [5] showed that a NHE1 protein that was truncated at amino acid 441 was not functional *in vivo* in mice. The present study agrees with this result.

Other early studies have examined various characteristics of the NHE1 protein with alterations in the length of the cytoplasmic regulatory domain. Wakabayashi [35] initially demonstrated that the C-terminal cytoplasmic regulatory domain determines or modifies the set point of the Na^+^/H^+^ exchanger mediating growth factor signals. They made a mutant that was truncated at amino acid 515 and found approximately 25% of activity remained. This was somewhat higher than what we found with the 543-NHE1 protein, but within a comparable range. In their study [35] it should be noted that they used transient transfections and did not correct for the expression levels or targeting of NHE1 protein with a tag, so levels of activity per amount protein on the cell surface are not comparable.

Two other studies characterized truncation mutants of NHE1. One [36] examined the osmotic responsiveness of truncated NHE1 mutants. They found that hypertonic stimulation of NHE1 was maintained if constructs truncated at amino acids 698 or 703, while termination at amino acid 635 eliminated much of the response to osmotic challenge. Our study did not examine responses to osmotic challenge though it would seem likely that the 735-NHE1 protein is likely functional in this regard and that the shorter constructs are not. They also noted that a mutant truncated at amino acid 635 behaved as if constitutively activated, which contrasts with the 735-NHE1 protein. Wakabayashi [35] also found that a NHE1 protein truncated at amino acid 792 had greater activity than the wild type protein. The reason for the discrepancy is not clear at this time however a truncation at amino acid 635 would delete an auto-inhibitory calmodulin binding domain [37] possibly resulting in an activated NHE1 protein [36]. Large deletions of the distal region of the cytoplasmic tail may cause misfolding of the tail that may interfere with the function of the autoinhibitory domain accounting for some of the discrepancy in results. As noted above, distal regions of the NHE1 tail have been shown to interact with more proximal regions of the tail domain [33].

Another early study [38] analyzed the effect of a series of tail deletions and suggested that the tail be divided into four domains of varying sensitivity to internal pH, consisting of 516–595, 596–635, 636–659 and 660–815. Their assays showed little effect of deletions in domain IV (amino acids 660–815), which overlaps with the 735-NHE1 mutation of the present study. It must be remembered however that correction for surface processing and targeting were not possible at that time. Additionally, pHi measurement was made using either [^14^C] benzoic acid or ^22^Na which do not allow for measurement of the changes in internal pH over short periods of time and does not allow measurement of times to recovery to resting pHi. Several studies [27, 28, 31, 39] since these earlier ones have shown that mutations to individual amino acids within this more distal domain, affect NHE1 activity and regulation confirming that this region has an important modulatory role on the membrane domain. Additionally, the regulation of NHE1 by amino acids in this tail domain may vary in different cell types and under different cell conditions [40, 41] which might account for different results in the early studies.

We have previously demonstrated that a mutation in the membrane domain of human NHE1 results in a protein with no detectable activity [42]. In this case a mutation of an amino acid near the cation binding site of the protein may have disrupted transport. Additionally, we also recently demonstrated that a mutation in the regulatory tail of NHE1 results in defective regulation of the NHE1 protein [43]. Taken together with the results of the present study, it appears that polymorphisms in the NHE1 can lead to significant detrimental effects on NHE1 protein function. As demonstrated by deletion or disruption of the NHE1 gene [6], significant downstream effects in humans can result. While no records were kept of alterations in the phenotype of mutations discovered in the 1000 genome project, it seems likely that SCP’s would have significant manifestations, especially on those mutations that result in a totally inactive protein in homozygotes. Future studies will investigate these effects.

## Supporting Information

S1 Fig Immunocytochemical localization of NHE1 in CHO and AP1 cells. Upper row, CHO cells which contain their own endogenous NHE1 protein. Bottom row, AP1 cells (that do not have an endogenous NHE1 protein) that were stably transfected with the 735 stop NHE1 protein. Column 1, DAPI staining of cells. Column 2, cells were reacted with anti-NHE1 antibody that reacts with the distal region of the NHE1 cytosolic tail. Column 3, merge of the images of the first two columns. Anti-NHE1 protein reacts with the endogenous, full length NHE1 protein present in wild type CHO cells. There was no signal in the AP1 cells transfected with the 735 stop NHE1 protein. AP1 cells have no endogenous NHE1 protein. Since the 735 stop NHE1 protein is shortened, the monoclonal anti NHE1 antibody site is absent and the antibody does not react with the 735 stop protein.
(PDF)
