# Supplementary material for: Stop Codon Polymorphisms in the Human SLC9A1 Gene Disrupt or Compromise Na+/H+ Exchanger Function
Source: PLoS One. 2016 Sep 16;11(9):e0162902. doi: 10.1371/journal.pone.0162902 (PMC5026351; doi:10.1371/journal.pone.0162902)
Supplement: S1 Fig — Upper row, CHO cells which contain their own endogenous NHE1 protein. Bottom row, AP1 cells (that do not have an endogenous NHE1 protein) that were stably transfected with the 735 stop NHE1 protein. Column 1, DAPI staining of cells. Column 2, cells were reacted with anti-NHE1 antibody that reacts with the distal region of the NHE1 cytosolic tail. Column 3, merge of the images of the first two columns. Anti-NHE1 protein reacts with the endogenous, full length NHE1 protein present in wild type CHO cells. There was no signal in the AP1 cells transfected with the 735 stop NHE1 protein. AP1 cells have no endogenous NHE1 protein. Since the 735 stop NHE1 protein is shortened, the monoclonal anti NHE1 antibody site is absent and the antibody does not react with the 735 stop protein. (PDF) [file pone.0162902.s001.pdf]

Supplementary Fig. 1

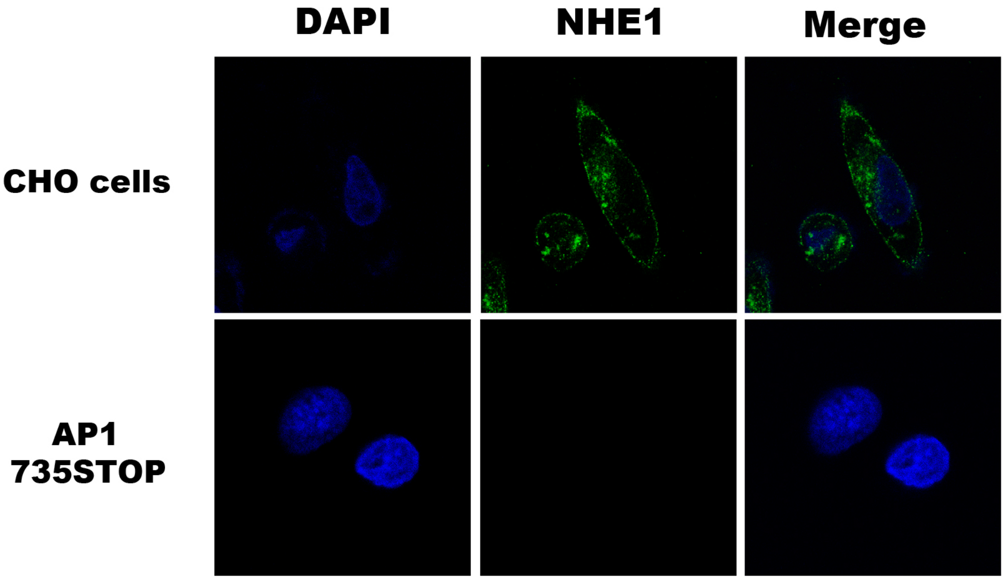

Fig. S1. Immunocytochemical localization of NHE1 in CHO and AP1 cells. Upper row, CHO cells which contain their own endogenous NHE1 protein. Bottom row, AP1 cells (that do not have an endogenous NHE1 protein) that were stably transfected with the 735 stop NHE1 protein. Column 1, DAPI staining of cells. Column 2, cells were reacted with anti-NHE1 antibody that reacts with the distal region of the NHE1 cytosolic tail. Column 3, merge of the images of the first two columns. Anti-NHE1 protein reacts with the endogenous, full length NHE1 protein present in wild type CHO cells. There was no signal in the AP1 cells transfected with the 735 stop NHE1 protein. AP1 cells have no endogenous NHE1 protein. Since the 735 stop NHE1 protein is shortened, the monoclonal anti NHE1 antibody site is absent and the antibody does not react with the 735 stop protein.
